# Fatal Disseminated Hepatitis E in an Adult Patient with *IKBKB* GOF Mutation

**DOI:** 10.1007/s10875-024-01721-w

**Published:** 2024-05-17

**Authors:** Leif G. Hanitsch, Marion Muche, Helena Radbruch, Jörg Hofmann, Victor M. Corman

**Affiliations:** 1 Institute of Medical Immunology, Charité - Universitätsmedizin Berlin, Corporate Member of Freie Universität Berlin and Humboldt Universität zu Berlin, Campus Virchow, Augustenburger Platz 1/ Südstraße 2, 13353 Berlin, Germany; 2 Berlin Institute of Health at Charité - Universitätsmedizin Berlin, BIH Center for Regenerative Therapies (BCRT), Berlin, Germany; 3 Department of Gastroenterology, Infectious Diseases, and Rheumatology (Campus Benjamin Franklin), Charité - Universitätsmedizin Berlin, corporate member of Freie Universität Berlin and Humboldt-Universität zu Berlin, Berlin, Germany; 4 Department of Neuropathology, Charité-Universitätsmedizin Berlin, Corporate Member of Freie Universität Berlin and Humboldt-Universität zu Berlin, Berlin, Germany; 5 Labor Berlin - Charité Vivantes GmbH, 13353 Berlin, Germany; 6 Institute of Virology, Charité-Universitätsmedizin Berlin, Corporate Member of Freie Universität Berlin, Humboldt-Universität zu Berlin, Berlin Institute of Health, Charitéplatz 1, 10117 Berlin, Germany; 7 German Centre for Infection Research (DZIF), Partner Site Charité, Berlin, Germany

To the Editor

Homozygous loss-of-function mutations of the *IKBKB* gene, encoding inhibitor kappa B kinase 2 (IKKβ or IKK2) protein, are a known cause for autosomal recessive severe combined immunodeficiency (SCID) albeit with low levels of immunoglobulins but relatively normal T and B cell counts [1]. Heterozygous gain-of-function (GOF) mutations in the same gene have been reported to cause an autosomal dominant hypomorphic combined immunodeficiency disorder with moderate lymphocytopenia and hypogammaglobulinemia. Clinical symptoms have so far been characterized by recurrent sinopulmonary infections, immune dysregulation and epithelial defects with a cutaneous phenotype [2, 3].

We present the case of a fatal disseminated hepatitis E infection in a 52 year old female caucasian patient of German residence with *IKBKB* mutation. The patient was diagnosed with chronic hepatitis E in 2017 without previous hepatic diseases. Apart from one herpes zoster infection 10 years earlier and a stable erythematotelangiectatic rosacea stage I-II with central facial distribution since early adulthood, the patient did not show an increased susceptibility to infections, autoimmunity, lymphoproliferation or other relevant medical conditions. As chronic hepatitis E is known to occur in immunosuppressed individuals such as transplant patients, immunological work-up was initiated.

Immunological work-up revealed normal IgG, IgA and IgM levels but a combined IgG2/IgG4-subclass deficiency and non-detectable antibodies against pneumococcal vaccine (specific pneumococcal-IgG remained below detection levels after sequential conjugated and polysaccharide vaccination). The patient was a known hepatitis B non-responder. HEV-IgG was detectable as well as specific IgG antibodies against tetanus toxoid and diphteria toxoid. In addition, lymphocyte phenotyping showed significantly reduced CD4 + and CD8 + T cells (150/µl and 120/µl respectively) with diminished proportion of CD45RA + CD4 + naïve T cells of 1–2%. CD19 + B cells had normal counts but expressed greatly decreased percentage of class-switched memory B cells (0,3% of CD19 + B cells) (see Table 1). Whole exome sequencing identified a previously reported heterozygous mutation in *IKBKB* (c.607G > A; p.V203I) (cDNA.793G > A, g.37639G > A). No other probable germline or somatic mutation was identified. Segregation analysis of the patient’s non-consanguineous parents was not possible. Type I interferon (IFN) autoantibodies (IFN alpha and IFN omega) were undetectable.

Off-label treatment with ribavirin was without clinical benefit and viral copy load of hepatitis E virus (HEV) persisted at a high level (> 5.000.000 IU/ml). Our patient was started on immunoglobulin substitution therapy. However, persistently elevated viral load of HEV required repeated treatment with ribavirin alone or in combination with interferon. Treatment was stopped due to severe hematopoetic adverse events requiring weekly transfusions and treatment with Thrombopoetin receptor agonist Eltrombopag (see supplemental table S1).

Viral load continued to remain high and the clinical condition deteriorated. In 09/2019 the patient was admitted to hospital due to acute on chronic liver failure. New neuro-psychological symptoms were first regarded as hepatic encephalopathy. In the absence of hyperammonemia further diagnostic work-up was conducted and revealed a positive PCR for HEV in cerebrospinal fluid (CSF). No other pathogens were detected by Multiplex PCR in CSF. Patient was admitted to ICU with clinical symptoms of septic shock and tonic-clonic seizure. Our patient deceased shortly thereafter without identification of other pathogens.

Post-mortem autopsy revealed liver cirrhosis without any other relevant organ pathology including no signs of neuro-inflammation or –degeneration. To assess the role of HEV we further analyzed different tissue samples from duodenum, liver and medulla oblongata and tested for the presence of HEV. HEV RNA and HEV specific RT-qPCR was conducted [4] and was positive in all tissue types. Interestingly, we observed differences in the consensus sequence in different tissues suggesting independent viral replication and a rare case of disseminated hepatitis E infection and compartmentalization.

We describe a case of a chronic hepatitis E with disseminated and fatal outcome in an adult female patient with no previous signs for an immunocompromised state and consequently diagnosed a heterozygous gain-of-function (GOF) IKBKB variant. The variant has undergone functional testing before and NFKB activation was shown by others [2].

Hepatitis E virus (HEV) is a hepatotropic RNA virus that is a rare cause of acute liver failure and usually does not lead to chronic infections in healthy individuals. Of four genotypes, genotype 3 is almost universally the cause in Europe, and the genotype does not influence the propensity to chronic infection. Impaired HEV epitope-specific CD4^+^ and CD8^+^ T cell responses are associated with the development of chronic hepatitis E [5], a defect that may explain the chronic infection in our patient. Chronic HEV is well-described in patients with solid organ transplantation, human immunodeficiency virus patients with low CD4 counts, in patients with hematological malignancy and in those receiving systemic chemotherapy.

Considering the high prevalence of hepatitis E with an IgG-seropositivity rate of approximately 10–20% in the general population, the underlying pathomechanisms of chronic hepatitis E infection remain poorly defined. So far, no other cases of chronic hepatitis E have been reported in *IKBKB* GOF patients. It remains to be determined, whether the chronic and fatal disease course is related to the mutation in *IKBKB*. However, other known risk factors for a chronification of hepatitis E were not identified.

A limitation to our reported case is that we did not assess T cell specific immunity to HEV. In addition, we never had the opportunity to conduct an in-depth immunological phenotyping before the onset of the chronic hepatitis E infection. In particular, the repeated treatment with ribavirin is very likely to have affected the hematopoietic compartment. Similarly, we could not identify additional autoinflammatory aspects in our patient, as was described previously [3].

Our findings highlight once again that all patients with unusual infections should be screened for an underlying immunodeficiency. Our case broadens the clinical picture of GOF mutations in *IKBKB*, showing that some individuals can present with a combined immunodeficiency later in adulthood with profound T-cell deficiency and impaired vaccination response, in this case leading to a disseminated and fatal hepatitis E infection.

**Table 1:** Clinical and immunological data of the patient

Age at diagnosis (in years)	48
Sex	female
Skin	Rosacea (since early adulthood)
Dental	normal
Recurrent respiratory tract infections	no
Recurrent gastrointestinal tract infections	no
other	Chronic Hepatitis E (since 47 yrs)
Bronchiectasis	no
lymphoproliferation	no
CNS	no (later HEV infection of CNS)
IgG	9.32 g/l (7–16 g/l)
IgG1	7.43 g/l (2,8–8,0 g/l)
IgG2	0.26 g/l (1,12 − 5,7 g/l) (-)
IgG3	1.37 g/l (0,24 − 1,25 g/l)
IgG4	< 0,0 g/l (0,052 − 1,25 g/l) (-)
IgA	2.65 g/l (0,7 − 4,0 g/l)
IgM	0.43 g/l (0,4 − 2,3 g/l)
IgE	0.8 kIU/l (< 100 kU/l)
Pneumococcal vaccine response	No response
Hepatitis B vaccine response	No response
CD3+	0,28/nl (0,9 − 2,2/nl) (-)
CD4+	0,15/nl (0,5 − 1,2/nl) (-)
CD4 + CD45RA + CCR7-	1% (> 10%) (-)
CD8+	0,12/nl (0,3 − 0,8/nl) (-)
CD8 + CD45RA + CCR7-	7% (-)
NK cells	0,19/nl (0,1 − 0,4/nl)
CD19+	0,16/nl (0,1 − 0,4/nl)
CD19 + IgD + CD27- (naïve)	88% (42,6–82,3%)
CD19 + IgD + IgM + CD27+ (MZ-like)	3.1% (7,4–32,5%) (-)
CD19 + IgD-IgM-CD27+ (class switched memory)	0.3% (6,5–29,1%) (-)
CD19 + CD21lowCD38low (activated)	0.4% (0,9 − 7,6%)
CD19 + CD21lowCD38 + + IgM+ (transitional)	0.5% (0,6 − 3,4%)
CD19 + CD21lowCD38 + + IgM- (switched plasmablasts)	0.1% (0,4 − 3,6%) (-)

### Electronic Supplementary Material

Below is the link to the electronic supplementary material.

Supplementary Material 1

## Data Availability

The datasets generated during and/or analyzed during the current study are available from the corresponding author on reasonable request.

## References

[1] Pannicke U, Baumann B, Fuchs S, Henneke P, Rensing-Ehl A, Rizzi M (2013). Deficiency of innate and acquired immunity caused by an IKBKB mutation. N Engl J Med.

[2] Cardinez C, Miraghazadeh B, Tanita K, da Silva E, Hoshino A, Okada S (2018). Gain-of-function IKBKB mutation causes human combined immune deficiency. J Exp Med.

[3] Sacco K, Kuehn HS, Kawai T, Alsaati N, Smith L, Davila B (2023). A heterozygous gain-of-function variant in IKBKB Associated with autoimmunity and autoinflammation. J Clin Immunol.

[4] Melchert J, Radbruch H, Hanitsch LG, Baylis SA, Beheim-Schwarzbach J, Bleicker T (2023). Whole genome sequencing reveals insights into hepatitis E virus genome diversity, and virus compartmentalization in chronic hepatitis E. J Clin Virol.

[5] Suneetha PV, Pischke S, Schlaphoff V, Grabowski J, Fytili P, Gronert A (2012). Hepatitis E virus (HEV)-specific T-cell responses are associated with control of HEV infection. Hepatology.

