# Supplementary material for: Fatal Disseminated Hepatitis E in an Adult Patient with IKBKB GOF Mutation
Source: J Clin Immunol. 2024 May 17;44(5):120. doi: 10.1007/s10875-024-01721-w (PMC11101494; doi:10.1007/s10875-024-01721-w)
Supplement: Supplementary file 1 — Supplementary Material 1 [file 10875_2024_1721_MOESM1_ESM.docx]

Supplemental table S1: chronic hepatitis E disease course and treatments

|  | Treatment | Hepatitis E viral load (in IU/ml) | ALT  (norm < 31 U/l) |
| --- | --- | --- | --- |
| 09/2017 | none | 6,020,000 | 227 |
| 10/2017 | start 1^st^ cycle with Ribavirin 1g/day |  |  |
| 02/2018 | end 1^st^ cycle with Ribavirin |  |  |
| 03/2018 | none |  |  |
| 04/2018 | start 2nd cycle with Ribavirin 1g/day | 1,520,000 | 111 |
| 07/2018 | 2nd cycle with Ribavirin | 106,000 | 58 |
| 10/2018 | 2nd cycle with Ribavirin | 54,600 |  |
| 01/2019 | end 2nd cycle with Ribavirin | 270,000 | 55 |
| 04/2019 | none | 3,300,000 | 217 |
| 05/2019 | start 3^rd^ cycle Ribavirin 1.2g/day plus PEG interferonalpha 2a 180µg/week |  |  |
| 07/2019 | dose reduction due to side effects: Ribavirin 1g/day plus PEG interferonalpha 2a 90µg/week | negative | 14 |
| 11/2019 | end 3^rd^ cycle Ribavirin plus PEG interferonalpha 2a | 645 | 59 |
| 06/2020 | none | 482,000 | 294 |
| 12/2020 | none | 5,000,000 | 294 |
| 03/2021 | none | 5,000,000 | 164 |
| 10/2021 | none | 5,000,000 | 141 |
